# Electrical Stapedial Reflex Thresholds in Cochlear Implant Recipients with Cochlear Nerve Deficiency: A Retrospective Study

**DOI:** 10.1055/s-0045-1808276

**Published:** 2025-09-10

**Authors:** Megha Sasidharan, Anil Kumar, Pratik Agarwalla

**Affiliations:** 1 Department of Hearing Studies, Dr. S. R. Chandrasekhar Institute of Speech and Hearing, Bengaluru, India; 2 Department of Audiology, Dispur Hospital, Guwahati, India

**Keywords:** cochlear nerve deficiency, electrical stapedial reflex, post operative, children, cochlear implant

## Abstract

**Introduction:**

Cochlear nerve deficiency (CND) is characterized by a thin or absent cochlear branch of the vestibulocochlear nerve, leading to sensorineural hearing loss (SNHL). The effectiveness of cochlear implants (CIs) in patients with CND is debated, with some studies indicating poor outcomes and others showing limited speech detection and discrimination abilities. Accurate objective measures are essential for a successful CI mapping.

**Objective:**

To investigate the use of global stimulation with live speech to measure electrically evoked stapedial reflex thresholds (eSRTs) in CI recipients with CND and compare the results with behavioral comfort levels.

**Methods:**

This retrospective study reviewed the CI database from January 2015 to December 2023 to identify patients with CND. The eSRT measurements following CI were conducted using a standard procedure. These levels were compared with the behavioral comfort levels from their mapping data.

**Results:**

From the CI database of 273 recipients, six children with CND were identified whose eSRT levels were documented. Among these six, two children exhibited presence of electrical stapedial reflexes that correlated well with behavioral comfort levels in response to global stimulation of electrodes. None of them presented reflexes to individual electrode stimulation.

**Conclusion:**

Global stimulation of electrodes has the potential to elicit eSRT in patients with CND more than individual electrode stimulation. Further research is crucial to validate the effectiveness of global stimulation in eliciting eSRT in a larger cohort of patients with CND. This would be a feasible test as an outcome measure, and it would help provide better prognostic counselling.

## Introduction

Cochlear nerve deficiency (CND) is described as a small or absent cochlear branch of the vestibulocochlear nerve with the clinical manifestation of sensorineural hearing loss (SNHL) of unknown etiology and pathophysiology. Cochlear nerve deficiency is present in 2.5 to 21.2% of patients with congenital SNHL. 1 2 Inner ear malformations and CND, detectable with computed tomography (CT) and magnetic resonance imaging (MRI), contribute to 15 to 39% of pediatric SNHL cases. 3 4 In recent years, high-resolution MRI has been used to observe the neuromorphology and development of the internal auditory meatus (IAM) and cochlear nerve (CN). Researchers have proposed a new IAM nerve grading system and a CN classification system based on MRI findings, which are as follows: grades 0 to III indicated zero, one, two, and three nerve bundles observed in the IAM (aplasia); grade IV, four nerve bundles in the IAM with a hypoplastic CN (hypoplasia); and grade V, four nerve bundles in the IAM with a normal-sized CN and normal position of the nerves. 5 Cochlear implants (CIs) in patients with CND remain controversial, as some studies have reported very poor results, 6 while others have reported limited speech detection and discrimination.

Objective measures are crucial in defining the levels used for CI mapping. The association between CI outcomes and electrically evoked compound action potential (eCAP) measures has been evaluated in previous studies. 7 8 9 He et al. 10 reported eCAP being recorded at all test electrodes in children with normal-sized CN. In contrast, the eCAP could not be recorded at any electrode site in 4 out of 23 children with CND. For all other children with CND, the percentage of electrodes with measurable eCAPs decreased as the stimulating site moved in a basal-to-apical direction. In a similar study, researchers examined the eCAP thresholds and their correlation with the behavioral thresholds in the map for children with CND. 11 The findings indicated a significant correlation at the basal, middle, and apical electrodes, with the eCAP thresholds being either equivalent to or greater than the behavioral T-levels. Additionally, these thresholds were within the dynamic range of the map for ∼ 90% of the electrodes studied. 12

Another objective approach that could be useful in mapping is electrically evoked stapedial reflex threshold (eSRT). According to Raine et al. (1997), eSRT can be measured postoperatively using an immittance meter in the implanted or nonimplanted ear to gauge the response to electrical stimulation through the implant. 12 Intraoperatively, eSRT could be recorded by visually observing the stapedial contraction during surgery. The brainstem mediates the neuromuscular reaction known as the stapedial reflex. Jerger et al. (1988) found a link between eSRT and comfort levels (C or M levels) of a map and concluded that eSRT can be used to predict C levels. 2 13 Bresnihan et al. (2001) discovered that the eSRT approach consistently produced lower results than behavioral strategies, and that children who used map with eSRT wore their implant for longer periods of time with fewer instances of discomfort. 14 There is a strong association between CI programs developed using behavioral techniques and eSRT recording to assess C levels. 15 16 Thus, eSRT methods have become crucial in mapping protocol. Another study evaluated how well eSRT levels measured using different modes of stimulation to predict the clinically mapped M-levels of each patient's daily use program obtained behaviorally in the course of standard clinical care. 17 They identified progressively lower eSRT levels measured for 1-, 4-, and 15-electrode stimulation conditions in that order. Average eSRT levels measured with 15-electrode stimulation were closest to and not statistically different from the average clinically-based M-levels from the patients' clinical programs. Their results suggested that 1- or 4-electrode stimulation can be used to measure eSRT on a subset of electrodes, and 15-electrode stimulation can be used as an upper bound of stimulation during global adjustment of the speech coding map to a comfortable loudness level. However, global stimulation of the electrodes has not been much reported in the literature. The present study explored the use of global stimulation using live speech in eliciting electrical stapedial reflexes in individuals with CND fitted with CI.

## Methods

### Participants

The present retrospective study was conducted by reviewing the CI database in Custom Sound software, version 7.0 (Cochlear India Private Limited) from January 2015 to December 2023 to identify patients with CND. The patient records were confirmed through their medical history and imaging reports from the CI unit at the Department of Hearing Studies, Dr S R Chandrasekhar Institute of Speech and Hearing, Bengaluru, India. The study was approved by the Institutional Ethical Committee of Dr. S. R. Chandrasekhar Institute of Speech and Hearing with the number BSHRF/RC/IERC/IM/IS/O2/2024. Patient records were included using the following criteria: (1) diagnosed with bilateral sensorineural hearing loss with CND before implantation; (2) implanted with a Cochlear Nucleus device (Cochlear Ltd); (3) regularly programmed at our center. The exclusion criteria included: (1) children with any other malformations with or without CND. Table 1 shows the details of the demographics of the participants along with the eSRT and behavioral C levels (of basal electrode number 1 as reference) of patients with CND.

**Table 1:** Details of the participants

Patient nr.	Chronological age	Age of implant	Implant age	Implant	Implanted ear	MRI findings	C level (reference basal electrode number 1)	eSRT (reference basal electrode number 1)	Difference
1	6.8 years	2.5 years	4.3 years	CI24RE (CS)	Right	Bilateral cochlear nerve deficiency	203	Absent	NA
2	3.4 years	2.2 years	1.2 years	CI24RE (CS)	Right	Bilateral cochlear nerve deficiency	177	Absent	NA
3	4.8 years	3.1 years	1.7 years	CI24RE (CA)	Left	Atretic bilateral internal auditory canals with hypoplastic vestibulo-cochlear nerves and normal 7 ^th^ nerves	182	Absent	NA
4	6.9 years	4.8 years	2.1 years	CI24RE (CA)	Right	Agenesis of bilateral 8th cranial nerve, normal 7 ^th^ cranial nerve	137	138	-1
5	1.2 years	1.0 years	2 days	CI24RE (CA)	Right	Hypoplastic right, absent left 8th cranial nerve, normal right and left hypoplastic 7 ^th^ cranial nerve with aberrant course, coursing antero- Medial & superior to common cavity	197	201	-4
6	4.2 years	4.0 years	1.2 months	CI422	Left	Aplasia of right and hypoplasia of left 8 ^th^ cranial nerve, normal 7th cranial nerve	160	Absent	NA

**Abbreviations:** MRI, magnetic resonance imaging; NA, not applicable as eSRT is absent.

### Behavioral C Levels

All the mapping sessions were done by a trained audiologist with the Custom Sound Software Version 5.1 (Cochlear Ltd). Prior to eSRT measurement, the behavioral responses were measured by selecting 5 electrodes (1, 6, 12, 16, 22) and interpolating them. Here, stimulation began at 5 CL above the previously measured behavioral C levels. If the child showed no response, then the level of stimulus was raised by 5 programming units, and the response was observed. If the child showed discomfort, three programming units were reduced and then increased or decreased by one programming unit until the audiologist perceived that the patient was hearing comfortably.

### eSRT Measurements

The eSRT measurements postcochlear implantation were performed using standard procedure by trained audiologists in the CI unit at the institute. A GSI Tympstar or GSI Tympstar Pro middle ear analyzer (Grason-Stadler, Inc.) was used for tympanometry. Participants having a 'type-A' tympanogram curve underwent eSRT. A 226-Hz probe tone was introduced into the target ear to record the stapedius reflex, and the eSRT was obtained using an implant speech processor coupled to an HP (Hewlett-Packard Enterprise) or a Fujitsu (Fujitsu Limited) laptop with custom sound software.

**Fig. 1:**
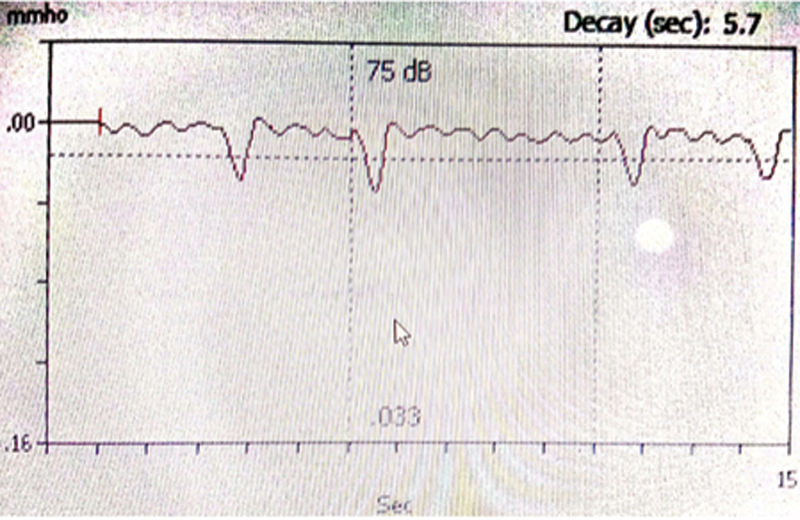
Presence of time-locked deflection of the reflex with the presentation of live speech stimuli in reflex decay mode of immittance instrument.

In the programming software, the option “live” was chosen, which allowed the participant to hear live speech. The response was measured by presenting live speech at a conversational level. The stimuli used to elicit reflexes were monosyllables spoken by the implant audiologists at a moderate level at three inches from the processor microphone. The eSRT responses were collected within the same time window using manual reflex decay for 15 seconds. The stimulation was started at 5 programming units above the previously mapped behavioral C levels. If no reflex was seen, then the current level was raised by 5 programming units. If a clear and time-locked downward deflection was present, the stimulus level was decreased by 2 programming units until no deflection was observed. Then, 1 programming unit was increased or decreased to derive the accurate value. The lowest stimulation level at which a deflection was obtained on three repeated presentations was considered as eSRT. Fig. 1 depicts the deflection as seen during the measurement of eSRT in a CI recipient. The increase in stimulation level was restricted to not more than 10 programming units above the participants' behavioral C levels. If the child showed any sign of discomfort, the testing was terminated.

## Results

From the CI database, a total of 273 recipients were identified, among whom 6 children had CND whose eSRT levels were recorded. All 6 CI recipients with CND underwent global stimulation of the electrodes using speech stimuli through Custom Sound Software to measure their eSRT. Among the six, two individuals had electrically-evoked stapedial reflexes. The current levels at the basal electrodes were chosen as a reference to compare the C levels and eSRT levels. Global stimulation yielded reflexes at 138 and 201 current levels, respectively, for the two recipients. For both children, the behavioral C levels across the electrodes were lower than the eSRT thresholds obtained through global stimulation, as shown in Table 1 . According to radiographic findings, the first child (Patient number 4) had bilateral agenesis of the 8th cranial nerve, and the second child (Patient number 5) presented with hypoplastic right and absent left 8th cranial nerve. Activation of individual electrodes with pulsatile stimuli had not produced reflexes in any of the participants.

## Discussion

Cochlear implantation in children with CND, particularly those with CN aplasia, has been contentious. Patients with CND would have limited nerve stimulation from electrical impulses given by CI electrodes. In turn, this would reduce the amount of neurological activity produced at higher centers along the auditory pathway and its related regions. Thus, in theory, the benefit of CI may be compromised if the CN is absent or hypoplastic. However, successful implantation outcomes show that even children with CND can benefit from CI. 5 18 19 20 21 22 Electrically-evoked stapedial reflex threshold is one objective tool to measure the outcome of CI, and the current study explored the use of eSRT in CND.

In the current study, CND was identified in 2% of the CI recipients from the database. This is in accordance with the literature, which reports retrospective findings of CI recipients. 23 The research has noted a strong relationship between eSRTs and comfort levels determined by subjective assessments. 13 24 25 26 In the current study, two out of the six patients presented with eSRT, and the difference between the eSRT and behavioral C levels was in accordance with the literature. Thus, when the stapedius reflex can be identified postoperatively, the eSRT may be effective for mapping speech processor, 19 which can be applicable for patients with CND. Most of the studies are performed using pulsatile stimulation of individual electrodes, which could be the reason for the lack of reports on eSRT in CND. On the other hand, it has been speculated that global stimulation of electrodes using live speech would increase the chances of eliciting a reflex. This is the same principle applied to acoustic reflex thresholds, which are lower in response to broadband stimulation. 27 Charoo et al. supported this speculation through their study, which showed a direct relationship between the number of electrodes stimulated and the M levels obtained behaviorally. In their study, there was no statistical difference between the eSRT obtained through 15 electrode stimulation and the M levels of the patient's map. 17 Although there was not enough data to perform a statistical measure in the current study, global stimulation of the electrodes using live speech elicited reflexes in 2 out of 6 patients, whereas individual electrode stimulation of apical, medial, and basal electrodes elicited no eSRT in any patient.

Radiologic indicators, including IAM diameter, bony cochlear nerve canal patency, cochlear aperture status, and cochlear anatomy, cannot reliably predict whether the CN is present or absent. Even though a diameter of less than 1.5 mm can more frequently be connected to a lack of CN, CND can occur in both narrow and normal-sized IAMs. However, normal-appearing cochlea and IAM do not corroborate the presence of a CN sufficient for CI. 23 The MRI findings of the participants, as shown in Table 1 , are also contraindications for CI, but the candidacy was established in these patients using electrical auditory brainstem response (eABR). Thus, the presence of postoperative eSRT in the two patients is a positive indicator confirming electrical stimulation of the auditory nerve.

## Conclusion

Our study indicates that global stimulation of electrodes may be useful to elicit post-CI eSRT and supports its use in mapping CND over individual electrode stimulation. However, the study included a small sample of 6 patients, out of which eSRT was obtained for only 2 patients. Thus, further research is essential to validate the use of global stimulation versus individual electrode stimulation on a larger group of patients with CND. This would aid in appropriate rehabilitation measures as well as improved counselling on the prognosis.
